# Perception of Gamblers: A Systematic Review

**DOI:** 10.1007/s10899-020-09997-4

**Published:** 2021-03-04

**Authors:** Andrea Wöhr, Marius Wuketich

**Affiliations:** Hohenheim Gambling Research Center, University of Hohenheim, Schwerzstraße 46, 70593 Stuttgart, Germany

**Keywords:** Gambling, Gambling disorder, Perception of gamblers, Stigma, Problem gambler, Systematic review

## Abstract

It is generally assumed that gamblers, and particularly people with gambling problems (PG), are affected by negative perception and stigmatisation. However, a systematic review of empirical studies investigating the perception of gamblers has not yet been carried out. This article therefore summarises empirical evidence on the perception of gamblers and provides directions for future research. A systematic literature review based on the relevant guidelines was carried out searching three databases. The databases Scopus, PubMed and BASE were used to cover social scientific knowledge, medical-psychological knowledge and grey literature. A total of 48 studies from 37 literature references was found. The perspective in these studies varies: Several studies focus on the perception of gamblers by the general population, by subpopulations (e. g. students or social workers), or by gamblers on themselves. The perspective on recreational gamblers is hardly an issue. A strong focus on persons with gambling problems is symptomatic of the gambling discourse. The analysis of the studies shows that gambling problems are thought to be rather concealable, whereas the negative effects on the concerned persons‘ lives are rated to be quite substantial. PG are described as “irresponsible” and “greedy” while they perceive themselves as “stupid” or “weak”. Only few examples of open discrimination are mentioned. Several studies however put emphasis on the stereotypical way in which PG are portrayed in the media, thus contributing to stigmatisation. Knowledge gaps include insights from longitudinal studies, the influence of respondents‘ age, culture and sex on their views, the relevance of the type of gambling a person is addicted to, and others. Further studies in these fields are needed.

## Background

Gambling is increasingly perceived as normal leisure activity in most Western societies. A large part of the adult population participates in some sort of gambling such as lotteries or sports betting. Unlike many other leisure activities, gambling may however cause adverse impacts on the health and wellbeing of an individual and his/her environment. The concept of “gambling harm” summarises a wide spectrum of negative consequences such as financial problems, disruptions to work/study, damage to the health, emotional and/or psychological distress, deterioration in relationships, cultural harms and criminal activities (Browne et al. 2016). Due to its potential negative effects on health and well-being, harmful gambling can be placed in the same category as smoking, problematic alcohol and recreational drug use (Browne et al. 2020).^1^

One negative consequence of gambling is stigmatisation. Stigmatising behaviour has several functions; among others it stresses the difference between “normal” and stigmatised behaviour in an attempt to enforce norm-complying behaviour. Persons with gambling problems are often perceived in a negative light and exposed to stigmatisation (Carroll et al.; Hing et al. 2016b; Palmer et al. 2017). However, stigmatisation of people with gambling problems has so far received little attention in research (Hing et al. 2013). The few studies that have looked into it have clear findings (Dhillon et al. 2011; Hing and Russell 2017a; Miller and Thomas 2017a; Palmer et al. 2017; Peter et al. 2018). A number of persons with gambling problems internalises the negative views of the society, perceiving their problems as personal failure. This causes self-stigmatisation, extending into a downward spiral. Moreover, stigmatisation is a major therapeutic obstacle because affected persons are hesitant to reveal themselves and might generally withdraw from relationships (Brown and Russell 2019; Hing et al. 2013; Miller and Thomas 2017b).

It is assumed that not only problem gambling, but every form of (risky) gambling is to some extent stigmatised (Horch and Hodgins 2015). However, research so far has mostly focused on the stigmatisation of persons with gambling problems (Miller and Thomas 2017b).

In view of the situation just described, we aim to systematise empirical evidence on the perception of gamblers. To our knowledge, a systematic literature review on empirical studies investigating the perceptions of people who gamble or have gambling problems does not yet exist, although intensified research activities are needed in order to successfully counteract (self-)stigmatisation (Schomerus and Rumpf 2017).

## Method

### Initial Search and Study Selection

The research strategy was based on guidelines for conducting systematic literature reviews (Card and Little 2016; Cooper et al. 2009; Liberati et al. 2009; Rosenthal 2010). Data was collected from December 13 to 18, 2018 in three scientific databases. The databases Scopus, PubMed and BASE were selected to ensure a broad range of results, taking medical-psychological knowledge, social scientific knowledge and grey literature^2^ into account.

To determine the search terms, we collected 69 potentially relevant terms for the electronic search. After discussion of the individual keywords, the search strings were systematically reduced by the authors' evaluations of relevance for each term. The truncated “gambl*” (for: gambling) was then linked to one of the following search terms by an AND-condition: “Stigma*”, “public attitude”, “social attitude”, “devaluation", “discrimination”, “labelling”, “prejudice”, “rejection”, “responsibility “, “self-control”, “self-esteem”, “self-worth”, “shame”, “social distance” and “stereotyp*”. A separate search was performed for each of the search term pairs in each database. The literature search was only limited (inclusion criteria) in terms of language (English) and publication date (from 1980 onwards). This year was chosen as a starting point because pathological gambling was first included in the DSM-III in 1980. Any form of scientific knowledge (journal articles, grey literature, talks, etc.) was allowed.

**Fig. 1:**
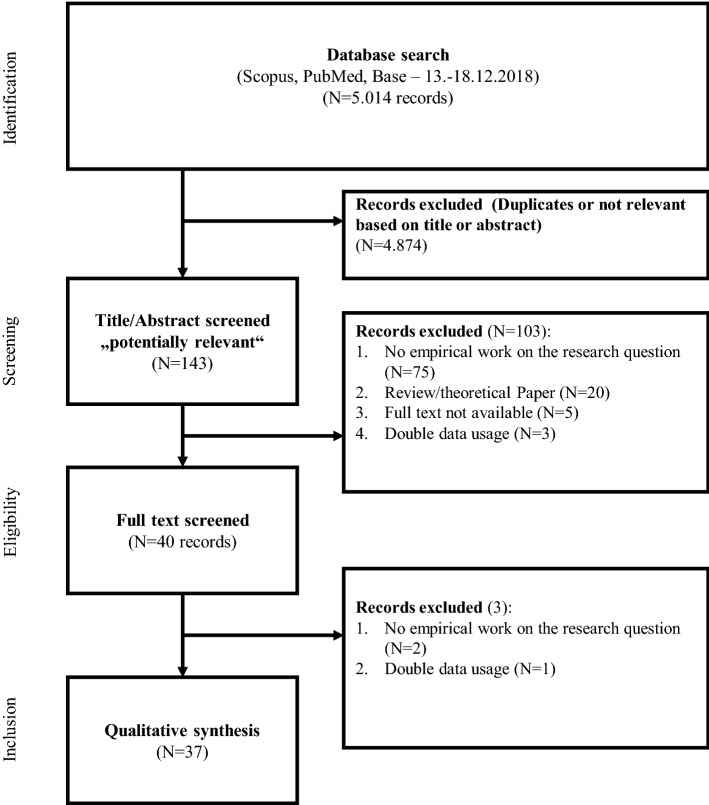
PRISMA flow-chart of the different phases of the systematic review

A total of 5.014 literature references was found. All individual database searches were exported and subsequently integrated, so that duplicates could be easily removed. As a next step, all literature references were screened on the base of title and abstract. Subsequently, 4.874 literature references could be removed, as they were not relevant to the review. Controversial cases were discussed and a joint decision was made. A large number of references could thus be removed as they were obviously off topic. 143 references were then classified as potentially relevant. By taking a closer look at the analysis and results section, more studies were removed. The remaining references were screened for eligibility by the two authors by reading the full text (*n* = 40). 37 of these citations have proved relevant and are included in the qualitative synthesis. The reasons for the exclusion are portrayed in Fig. 1.

### Systematisation of Literature and Contents

To systematise the relevant literature, the study characteristics were analysed in a descriptive way with focus on methodological and structural aspects such as the perspective (e. g. therapists on persons with gambling problems), type of sample (e. g. students, public), method of analysis (e. g. descriptive, content analysis) and country of origin of the sample.

Second, the content-related aspects were categorised. As a basis, the shared dimensional features of stigma proposed by Jones (1984) were used. Jones developed six categories to systematically describe stigma and to document the differences between stigmas. The category “concealability” describes the degree to which a stigma is visible to others. “Course” refers to the persistance of a stigma over time, “disruptiveness” to the degree to which a stigma interferes with social interactions. The category “aesthetics” refers to the potential to provoke a rejective attitude. “Origin” describes whether a stigma is believed to be inborn, accidental, or deliberate. Lastly, the category “peril” relates to the degree to which a stigma is understood as a personal menace or threat. The relevant literature was thoroughly searched to identify all aspects fitting into these categories. For practicability reasons, concealability and aesthetics were combined under the term of “concealability” because both dimensions basically refer to the visibilty of a condition.

Beyond Jones’ shared dimensional features of stigma, Hing et al. (2013) have developed further categories to describe the process of stigma formation. The category “labelling” describes the process of finding and assigning names or labels for certain human characteristics. The persons or groups to whom such labels are attached are identified with negative attributes and stereotypes (“stereotyping”). Consequently, the majority society distances itself from these persons or groups (“separation”). The differentiation between “the normal” and “the other” triggers a negative “emotional response”. When negative attitudes manifest in behaviour, the stigmatised persons experience a specific form of social exclusion (“status loss and discrimination”). As these categories build the reference for most current works either directly or indirectly (e. g. Brown and Russell 2019), they were also included in the present analysis.

## Results

### Study Characteristics

A total of 37 literature references (e. g. journal articles) was found. Some of these references contained several studies. E. g., an article may describe surveys obtained from different samples (e. g. from the general public, social workers and gamblers). Two publications each worked with the same sample but inquired on different topics (Hing and Russell 2017a and Hing and Russell 2017b) or used different survey methods (Hing et al. 2015 and Hing et al. 2016d); consequently, they were also listed as separate studies. Altogether, 48 surveys were obtained. The chosen method has the advantage of portraying each individual study separately, implicitly accepting that certain samples attain more weight.

Only one study (Crawford et al. 1989) was published before the beginning of the new millennium. Most of the studies were published after 2010. Apparently, the issue has not played a significant role in scientific debate before.

In terms of analytic methods and samples, the studies were very heterogeneous. Tables 1, 2 and 3 portray relevant methodological and structural characteristics of all studies. 17 studies used a qualitative, 26 a quantitative methodology. Five studies used both methodological approaches. Of the 17 qualitative studies, nine used conventional personal in-depth interviews, three used focus groups, two discourse analyses and three film analyses. 20 out of 26 quantitative studies conducted an online survey. The remaining six studies consisted of vignette studies (*n* = 5) and one corpus-based text analysis. Almost exclusively, cross-sectional surveys were carried out. Only one film analysis (Chan and Ohtsuka 2011) could be categorised as longitudinal study. The large variety of methods challenged the synthesis of the results obtained in these studies.

**Table 1:** Qualitative studies

Study	Origin	Perspective (PG = persons with gambling problem)	Sample	Recruitment/Source	Mean age (in years)	Proportion of women	Data analysis
*Interviews*							
Miller and Thomas (2018)	AU	PG on "responsible gambling"-discourse	26 EGM players with experience of gambling problems who are also involved in peer support and advocacy organisations	Peer support and advocacy organisations, online and email strategies, word of mouth	53	57.7%	Semiconstant comparative method
Miller and Thomas (2017b)	AU	Gamblers on various aspects of gambling	100 Gamblers	Social media, local newspapers, direct recruitment, word of mouth	38.2	38%	Constant comparative method
Hing et al. (2016b)	AU	PG on PG	44 People experiencing gambling problems	Through a prior survey	n.a.	36.4%	Interpretive phenomenology
Hing et al. (2016a)	AU	Gambling councellors on PG	9 Councellors	Email to gamblers’ help agencies	n.a.	71.4%	Interpretative phenomenological analysis
Li et al. (2014)	AU	Youths on their own gambling behaviour	15 Youths with Chinese origin experiencing gambling problems	Snowball system	25.7	28.6%	Thematic analysis
Carroll et al. (2013)	AU	Service providers on PG	35 Health service providers	Problem gambling services	n.a.	n.a.	Grounded theory analysis
Carroll et al. (2013)	AU	EGM gamblers on PG	25 High-intensity players of EGM	Problem gambling services	n.a.	n.a.	Grounded theory analysis
Carroll et al. (2013)	AU	PG on PG	30 Clients at health and gambling service providers and from a previous survey	Contact to problem gambling services, from a previous survey	n.a.	n.a.	Grounded theory analysis
Carroll et al. (2013)	AU	Councellors on PG	38 persons with gambling problems and financial councellers	Conference attendees, contact to problem gambling services	n.a.	n.a.	Grounded theory analysis
*Focus group interviews*							
Egerer (2013)	FI	Social workers on PG	31 Social workers	Advertisements in professional journals, contact to social offices	n.a.	87.1%	Semiotic analysis
Egerer (2013)	FR	Social workers on PG	27 Social workers	Advertisements in professional journals, contact to social offices	n.a.	96.3%	Semiotic analysis
Lopez-Gonzalez et al. (2018)	ES	PG on sports betting	43 Sports bettors experiencing gambling problems	FEJAR (Spanish Federation of Rehabilitated Gamblers)	33.2	0%	Thematic analysis
*Text/discourse analysis*							
Miller et al. (2016)	AU	Government and industry on "problem" and "responsible gambling"	Government and industry documents, television campaigns and warning signs	Government and gambling industry websites, television campaigns and RG materials	–	–	Thematic discourse analysis, constant comparative method
Miller et al. (2014)	AU	Press on problem gambling	339 Newspaper articles from the eight highest circulation newspapers in Australia	FACTIVA database	–	–	Content analysis, framing analysis
*Film analysis*							
Egerer and Rantala (2015)	Mixed	Portrayal of gamblers in films	72 Film scenes from 28 narrative fiction films from 1922 to 2003 about gambling in North American and West European mainstream cinema	–	–	–	Qualitative film analysis
Chan and Ohtsuka (2011)	HK	Portrayal of gamblers in films	11 Hongkong films from 1952 to 2001 rated by 6 persons / n.a.	–	–	-	Qualitative film analysis
Sulkunen (2007)	Mixed	Portrayal of addiction in films	140 Scenes from 47 films portraying various addictions	–	–	–	Comparative group interviews using vignettes

**Table 2:** Quantitative studies

	Origin	Perspective (PG = persons with gambling problem)	Sample	Recruitment	Sample type	Mean age (in years)	Proportion of women	Data analysis
*Online questionnaire(s)*								
Gavriel-Fried and Rabayov (2017)	IL	Other	37 Individuals with gambling problems	Direct recruitment at rehabilitation treatment centers	Convenience	35	2.7%	Variance analysis
Hing and Russell (2017a)	AU	PG on public characterization of problem gambling	177 Persons with PGSI > 8 within last 12 months	E-mail to participants in previous surveys, Google advertisements	Convenience	40.3	33.5%	Correlation analysis, regression analysis, path analysis
Hing and Russell (2017b)	AU	PG on (anticipated) stigmatisation	177 Persons with PGSI > 8 within last 12 months	E-mail to participants in previous surveys, Google advertisements	Convenience	40.3	33.5%	Correlation analysis, regression analysis
Horch and Hodgins (2013)	CA	Students on PG	152 Students	University pool	Convenience	21.9	73.4%	Descriptive analysis, chi square analyses
Horch and Hodgins (2013)	CA	Students on PG	790 Students	University pool	Convenience	20.5	92%	Descriptive analysis, chi square analyses
Horch and Hodgins (2013)	CA	PG on PG	74 Problem gamblers	Newspaper ads	Convenience	41.5	32.5%	Descriptive analysis
*Chi square analyses*								
Koski-Jännes and Simmat-Durand (2017)	FI/FR	Professionals' beliefs about gambling and Internet addictions	520 Finnish and 472 French treatment professionals	E-mail to treatment providers	Convenience	n.a.	76 and 67% resp.	Descriptive analysis, variance analysis, logistic regression analysis
Lang and Rosenberg (2017)	US	Population: willingness to affiliate with persons with various addictions	612 U.S. residents between the ages of 18 and 65 years	Mechanical Turk	Convenience	n.a.	50%	Bivariate analysis, variance analysis
Gay et al. (2016)	AU	Influence of environment on emerging adult problem gambling	188 Emerging gamblers	Social media	Convenience	21.41	70.7%	Hierarchical regression analysis
Horch and Hodgins (2015)	CA	PG on PG	155 Adults with gambling problems	Newspaper advertisments, community posters	Convenience	42.3	31%	Binary logistic regression
Konkolÿ Thege et al. (2015)	CA	Population on various problem behaviours	4.000 Adults	Ipsos Canadian Online Panel	Representative	n.a.	64.3%	Descriptive analysis, bivariate analysis, discrimant function analysis, Pearson chi-square test
Feeney (2013)	US	Adults on problem gambling	Several surveys including 1.000 to 1.100 adults resp.	Ipsos’ Internet panel	Representative	n.a.	n.a.	Descriptive analysis
Koski-Jännes et al. (2012)	FI	Population: responsibility for addiction problems	740 Adults	Independent survey firm	Representative	45.6	52%	Descriptive analysis, variance and logistic regression analysis
Koski-Jännes et al. (2012)	FI	Professionals: responsibility for addiction problems	520 Professionals	Mail to in- and outpatient treatment centers and the Criminal Sanctions Agency	Convenience	41.8	76%	Descriptive analysis, variance and logistic regression analysis
Koski-Jännes et al. (2012)	FI	Clients: responsibility for addiction problems	78 Clients	Personal contact	Convenience	36.7	50%	Descriptive analysis, variance and logistic regression analysis
Cunningham et al. (2011)	CA	Public beliefs about gambling abuse	8.467 adults	Random digit dialing telephone survey	Representative	46.2	55%	Regression analysis
Arbour-Nicitopoulos et al. (2010)	CA	Adolescents' attitudes towards family members with a health problem	2.790 7th to 12th grade students	Ontario Student Drug Use and Health Survey 2007	Representative	15.1	n.a.	Logistic regression analysis
Blomqvist (2009)	SE	Population on various addiction problems	1.098 Adults	Mailed out by Statistics Sweden	Representative	44.2	49.6%	Factor analysis
Rockloff and Schofield (2004)	AU	Population: attitudes toward problem gambling treatment	1.203 Adults	Random-digit-dialed phone survey	Representative	45.8	49.7%	Descriptive analysis, factor analysis
Crawford et al. (1989)	UK	Attitude of population to various deviant groups	200 Adults	Door-to-door survey	Representative	n.a.	n.a.	Multiple regression analysis
*Vignette study*								
Palmer et al. (2018)	US	Population on various groups of gamblers (recreational, problem, etc.)	378 Adults	Mechanical Turk	Convenience	37.5	46.8%	Descriptive analysis, bivariate analysis, variance analysis
Peter et al. (2018)	US	Public stigma associated with casino gambling, Internet gaming, and eSports gambling problems	504 adults	Mechanical Turk	Convenience	37.2	50.6%	Descriptive and variance analysis
Hing et al. (2016d)	AU	Population on problem gambling compared to other health conditions	2.000 Adults	Online panel	Representative	46	51.5%	Repeated measures analysis, multiple regression analyis
Dhillon et al. (2011)	CA	Students of East Asian and Caucasian ancestry on East Asian or Caucasian problem gamblers	n = 50 with Caucasian background, n = 64 of East Asian ethnicity	Psychology Department Research Participation System (RPS) at the University of Calgary	Convenience	20.8	71.1%	Variance analysis, bivariate analysis
Horch and Hodgins (2008)	CA	University students and stigmatisation of different health conditions	249 Students	Research Participation System (RPS) at the University of Calgary	Convenience	20.8	52.6%	Descriptive analysis, exploratory moderator analysis, variance analysis
*Text analysis*								
Leung (2016)	SG	Representation of gamblers in Singapore newspaper texts	889 Articles from the daily paper The Straits Times	LexisNexis	–		–	Corpus analysis, collocation analysis, critical discourse analysis

**Table 3:** Qualitative and quantitative studies

Study	Origin	Perspective (PG = persons with gambling problem)	Sample	Recruitment	Sample type	Mean age (in years)	Proportion of women	Data analysis
Hing et al. (2015)	AU	Adults on problem gambling	2.000 Adult residents of Victoria	Online panels	Representative	n.a.	n.a.	Quantitative: correlation analysis, regression analysis
Hing et al. (2015)	AU	PG on (perceived) stigma	203 Adults with PGSI > 8 during the last three years	Email to gamblers' help agencies	Convenience	40.9	33.5%	Quantitative: descriptive analysis
Hing et al. (2015)	AU	PG/councellors on (perceived) stigma	44 gamblers; 9 councellors	Email to gamblers' help agencies and from previous survey	Convenience	n.a.	36.4%; 22%	Qualitative: interpretative phenomenological analysis
Grunfeld et al. (2004)	CA	Professionals on PG	Discourse within the professional online forum "Gambling Issues International"	–	–	n.a.	n.a.	Unobtrusive observation; computer-based content analysis of online posts
Grunfeld et al. (2004)	Mixed	Professionals' concern about stigmatisation of PG	39 Professionals	Platform (gambling listserv for professionals)	Convenience	n.a.	n.a.	Questionnaire

With respect to their origin, 40 from the total of 48 studies were journal articles, which, owing to the peer review process, usually guarantee a higher quality of results; although even there the quality of many of the studies could be considered as low as e. g. descriptions as to the methods used were missing. Seven (Carroll et al. 2013; Hing et al. 2015) studies stemmed from grey literature; one was a presentation (Feeney 2013). One of the grey literature sources, namely the research report by Hing et al. (2015), encompasses three very detailled studies described on a total of 282 pages and will take up ample room in the following chapters.

The vast majority of studies were carried out with either Australian (*n* = 19) or Canadian samples (*n* = 10), which might impact the generalisability of the results of the present study. Four studies were carried out with Finnish and four with US samples. One study each was conducted in France, Spain, Sweden, the United Kingdom and Israel. Two studies were based on international mixed samples. Two out of the three film analyses took an international perspective, whereas Chan and Ohtsuka (2011) considered only Hong Kong films. In addition, there was a corpus-based text analysis of newspaper articles from Singapore (Leung 2016).

Representative samples were clearly outnumbered by convenience samples, which also affects the overall quality of the results. Population representative studies were available for Australia, Canada, Finland, Sweden and the UK. Taken together, more females were interviewed in the studies than men, which is why the following tables identify the proportion of females in the samples.

### Perception of Gamblers

Stigmatisation of gambling and gamblers is a comparatively new topic for research. In order to get an impression of the dimension of the issue, several studies compared the degree of stigmatisation for gamblers with the degree of stigmatisation for persons with various substance and non-substance use disorders, mental health problems and/or other conditions. In addition, this information might be useful during the development of health policy measures. For example, comparable stigma reduction strategies from other areas (e. g. mental illness) could be adopted for the gambling field.

In general, drug use was more heavily stigmatised than alcohol abuse (Arbour-Nicitopoulos et al. 2010; Feeney 2013), alcohol abuse more than problem gambling (Arbour-Nicitopoulos et al. 2010; Feeney 2013; Hing et al. 2015; Horch and Hodgins 2008). Mental health problems were less stigmatised than problem gambling (Arbour-Nicitopoulos et al. 2010; Feeney 2013); however, persons with schizophrenic disease were more heavily stigmatised than persons with gambling problems (Hing et al. 2015; Horch and Hodgins 2008).

Stigmatisation levels were higher for persons who were officially diagnosed as disordered gamblers (Palmer et al. 2018). Self-stigmatisation in persons with gambling problems increased with age, female sex, lower self-esteem, higher problem gambling severity scores, and use of secrecy as a coping mechanism (Hing and Russell 2017b).

Not all forms of gambling were equally stigmatised. Sports bettors were less heavily stigmatised than other gamblers (Lopez-Gonzalez et al. 2018). EGM gamblers were seen and desribed in a particularly negative way (Miller and Thomas 2017b). This could be related to the fact that in some games of chance, the gamblers’ skills play or at at least attributed a greater role. Apparently, gambling as an activity was not per se stigmatised, only problem gambling (Hing et al. 2015).

### Dimensions of Stigma

The following sections sum up the contents with reference to the dimensions of stigma as proposed by Jones (1984), as well as to the categories for the process of stigma formation as described in Hing et al. (2013).

#### Concealability

Most of the studies‘ respondents thought that it was easier to conceal problem gambling than alcohol use disorder or schizophrenia (Hing et al. 2015, 2016d). However, the majority also judged problem gambling to be as “at least somewhat noticeable” (Hing et al. 2015, 2016d) or “fairly noticeable” (Hing et al. 2016e). The fact that their condition seemed rather obvious to the public was not reflected by persons with gambling problems (Hing et al. 2015). This could lead to gambling disorder receiving less attention compared to other issues in society, such as drug and alcohol addiction.

#### Disruptiveness

When interviewed on the disruptiveness of problem gambling, most people indicated that problem gambling had at least a large effect on the affected persons‘ ability to work or study, to live independently and to be in a serious relationship (Hing et al. 2015, 2016d). Problem gambling was perceived to be “quite disruptive” (Hing et al. 2016e), “disruptive” (Hing et al. 2015) or “highly disruptive” (Hing et al. 2016d), even more than alcohol use disorder but less than schizophrenia (Hing et al. 2015). Persons with gambling problems seemed to anticipate that the public considered them to lead disruptive lifes, which in turn increased self-stigma (Hing and Russell 2017a).

#### Recoverability

Problem gambling was perceived to be recoverable by most respondents (Hing et al. 2015, 2016d, e), more so than schizophrenia (Hing et al. 2016d) and more (Blomqvist 2009) or similarly than alcohol use disorder (Hing et al. 2015). Change optimism was higher for persons with tobacco abuse but lower for persons using medical or illegal drugs (Blomqvist 2009). Untreated, recovery from problem gambling was thought to be easier than from mind altering substance addictions (Koski-Jännes et al. 2012); in the same vein, treatment was rated to be more important for “hard drugs” or alcohol than for gambling (Blomqvist 2009).

In Blomqvist (2009), the recoverability from problem gambling was rated high, whether with or without treatment. In Cunningham et al. (2011) and Feeney (2013), respondents were divided on whether it was possible for persons with gambling problems to recover without outside assistance. Professionals rated self-change as more difficult than lay persons (Koski-Jännes et al. 2012). Interestingly, there seem to be cultural differences: French professionals believed more in untreated recovery than their Finnish counterparts (Koski-Jännes and Simmat-Durand 2017). Compared to the general public, persons with gambling problems also rated treatment as less necessary (Cunningham et al. 2011).

#### Peril to Others and Self

Individuals with gambling problems are generally not perceived as being dangerous (Dhillon et al. 2011; Hing et al. 2016d; Peter et al. 2018). In Hing et al. (2015) problem gambling was thought to be “somewhat perilous”; but the respondents thought it unlikely that persons with gambling problems would cause peril to others and likeley that they would harm themselves.

Both in terms of peril to others and peril to self, persons with gambling problems were thought to be less dangerous than persons with alcohol use disorder or schizophrenia (Hing et al. 2015; Horch and Hodgins 2015). Interestingly, different types of games seem to be rated differently: Internet gamers were seen as less dangerous to be around than eSports gamblers or traditional casino gamblers (Peter et al. 2018).

#### Origin

The causes for gambling disorder were seen as rooted in personality traits, such as having an addictive personality (Carroll et al. 2013; Feeney 2013) or not enough willpower; lack of willpower might also imply that gambling habits of friends and relatives are taken over (Feeney 2013). Another issue mentioned was lack of control, discipline or even intelligence (Miller et al. 2014). In Horch and Hodgins (2008), bad character traits *and* stressful live events were seen as responsible for gambling disorder. Stressful life circumstances were identified as the most likely cause (Dhillon et al. 2011; Hing et al. 2015, 2016d, e). Finnish and French social workers seemed to think that the causes could be found in society rather than in the individual. Whereas the Finnish social workers regarded the individual as responsible for recovery, their French counterparts disagreed (Egerer 2013). In most studies however, the individual was thought to be responsible (Blomqvist 2009; Gay et al. 2016; Horch and Hodgins 2008, 2015; Konkolÿ Thege et al. 2015; Koski-Jännes and Simmat-Durand 2017; Koski-Jännes et al. 2012; Miller and Thomas 2017b).

In general, character flaws were viewed as being more associated with behavioral addictions than with substance use disorders (Konkolÿ Thege et al. 2015). Accordingly, the general public, professionals and the clients themselves thought that persons with gambling problems were responsible for their problem, more so than persons with substance use disorders (Koski-Jännes and Simmat-Durand 2017; Koski-Jännes et al. 2012).

### Emotional Reactions: Pity, Anger and Fear

Following Angermeyer and Matschinger (2003), three emotional reactions—pity, anger and fear—were measured in a number of studies. Mostly, the respondents indicated that they would feel pity towards persons with gambling problems, with some anger and some fear (Hing et al. 2015, 2016e). In Gay et al. (2016) and Horch and Hodgins (2008), respondents felt anger and pity towards problem gamblers equally or almost equally strong, but lower levels of fear. Both eSports gamblers and casino gamblers attracted more fear than the Internet gamer (Peter et al. 2018).

Respondents were more likely to pity persons suffering from schizophrenia than persons with gambling problems or with alcohol use disorder; the latter being at more or less the same level. In terms of anger, respondents felt as much anger against persons with gambling problems as against persons with alcohol disorder; again, persons with schizophrenia met with less anger. Respondents were more likely to fear persons with alcohol disorder and schizophrenia than persons with gambling problems (Hing et al. 2015).

### Dimensions of Stigma Creation

Dimensions of stigma creation include labelling, stereotyping, status loss and discrimination and social distancing (Hing et al. 2013).

#### Labelling

Although labels are necessary—e. g. for obtaining adequate treatment (Grunfeld et al. 2004)—they contribute substantially to the creation of stigma (Hing et al. 2016b; Peter et al. 2018). Several studies investigate on whether problem gambling was perceived as mental health disorder, physical health disorder, addiction, disease/illness, or as a diagnosable condition.

Gambling problems were either attributed to addiction (Hing et al. 2015) or equally to addiction and disease (Cunningham et al. 2011). Additionally, the majority of the respondents believed that problem gambling was a diagnosable condition (Hing et al. 2015). In an early study by Crawford et al. (1989), “compulsive gambling” was rated to be a disease rather than a “habit” or a “sin”.

Generally, the risk of becoming addicted to gambling was rated to be lower than to substances such as alcohol or drugs (Konkolÿ Thege et al. 2015; Lang and Rosenberg 2017); however, in Blomqvist (2009), it was rated to be slightly higher than to alcohol.

#### Stereotyping

Stereotypes of persons with gambling problems probably stem from culturally transmitted beliefs rather than from direct interactions and are therefore difficult to confront (Hing et al. 2016e). Frequent attributions included “irresponsible” (Hing et al. 2015, 2016e; Horch and Hodgins 2008, 2015; Miller and Thomas 2017b), “greedy” (Hing et al. 2015, 2016e; Horch and Hodgins 2013; Miller and Thomas 2017b), “antisocial” (Hing et al. 2015, 2016e; Horch and Hodgins 2013), “foolish” (Hing et al. 2015, 2016b, e), “impulsive” and “irrational” (Hing et al. 2015, 2016e; Horch and Hodgins 2013) and “untrustworthy” (Hing et al. 2015, 2016e; Horch and Hodgins 2015) on the part of the public.

Persons with gambling problems described themselves as “stupid” (Hing et al. 2015, 2016b; Miller and Thomas 2017b), “weak” or “losers” (Hing et al. 2015, 2016b). The gamblers also assumed that the general population judged them as “impulsive, irresponsible, irrational, anti-social, greedy, untrustworthy, unproductive, and deviant” (Hing et al. 2015), which is likely to increase self-stigma (Hing and Russell 2017a).

People with gambling problems also sensed that the media contributed to the formation of stereotypes by their emphasis on the negative consequences of gambling (Miller and Thomas 2017b, 2018) and on the responsibility of the individual (Miller et al. 2014). Persons with gambling problems were frequently portrayed as a deviant group, as opposed to the rest of the society, and they were perceived as having only themselves to blame (Leung 2016; Miller et al. 2016).

Studies focusing on the portrayal of gamblers in the movies described images of masculinity and coolness, self-control and the ability to enjoy oneself (Egerer and Rantala 2015). Only gamblers with limited abilities developed problems (Sulkunen 2007). However, the portrayal of gamblers has changed over the decades: from a sinner in the 50s over skilful and intelligent persons in the 80s to funny characters with a lack of moral in the 2000s (Chan and Ohtsuka 2011).

#### Status Loss and Discrimination

Alcohol disorder and schizophrenia were rated to be more likely to result in status loss than problem gambling (Hing et al. 2015). There was moderate agreement that a person would lose status or experience discrimination because of problem gambling, mostly in the fields of employment, child care and relationships (Hing et al. 2016e). Horch and Hodgins (2015) determined a discrimination value halfway between “seldom” and “sometimes”, suggesting that discrimination of individuals with gambling problems happens infrequently. Consequently, examples of discrimination were found to be rare (Hing and Russell 2017a), which was attributed to the concealability of problem gambling (Horch and Hodgins 2015) and the fact that many affected persons do not disclose their gambling problem. Negative responses included comments about wasting money or suggestions to do something better with life (Hing et al. 2016b).

#### Social Distance

Several studies investigated on the willingness of particpants to engage with persons with gambling problems. Compared to persons with alcohol disorder or schizophrenia, the respondents were less likely to distance themselves (Hing et al. 2015).

Most participants wished to keep distance or at least “some distance” to persons in this condition (Hing et al. 2015, 2016e; Lang and Rosenberg 2017; Rockloff and Schofield 2004), especially to persons whose condition had been officially diagnosed (Palmer et al. 2018). In other studies, the desire for segregation was found to be low (Gay et al. 2016; Horch and Hodgins 2008). Persons who were more familiar with persons experiencing gambling problems wanted less social distance (Dhillon et al. 2011). Creating more familiarity with the issue could help to develop effective strategies in the effort to confront stigma.

The desire for social distance was also influenced by the type of gambling. Participants were less willing to engage with casino gamblers than with e-sport gamblers, and more willing to engage with Internet gamblers than with e-sport gamblers (Peter et al. 2018).

## Discussion

The systematic review on studies dealing with the perception of gamblers revealed that research on this topic commenced to gather pace mainly after 2010, and was carried out primarily in Australia and Canada. Population representative studies were found for Australia, Canada, Finland, Sweden and the UK. Almost exclusively, cross-sectional surveys were carried out. In consequence, a change in public perception of gamblers could not be investigated. Neither were studies available that focussed primarily on effects of the respondents‘ sex and age, although this aspect was considered as one issue among others in some studies (e. g. Hing and Russell 2017b). As the vast majority of studies concentrated on problem gambling, conclusions on the perception of recreational gamblers could not be drawn. It would be of interest to have more and more comparable data from different countries and/or over time.

The analysis of the studies‘ contents showed that problem gambling was thought to be rather concealable. However, the negative effects on the concerned persons‘ lives were judged to be quite substantial. With respect to treatment and recovery, the opinions were divided. Most respondents seemed to think that the individual is responsible for finding a way out of the problem. The concept of problem gambling as an addiction does not seem to have found its way into the minds of the public. The provision of adequate information could be helpful.

Persons with gambling problems were described with a series of negative attributes. While some of these attributions have some basis in truth, others go well beyond the mark. This negative perspective was mirrored in the gamblers’ negative picture of themselves, as a sign of self-stigmatisation. The way in which persons with gambling problems are portrayed in the media helps to aggravate this process. This could be another starting point for countering stigmatisation.

Only few examples of open discrimination of persons with gambling problems were mentioned, probably because the condition can be concealed more easily than other addictions. The general public wanted to keep at least some distance to persons with gambling problems; the desire for distance however diminished with increased familiarity. This knowledge can be used, for example, for public awareness campaigns that show that “ordinary people” can develop gambling problems. As one study (Peter et al. 2018) showed, the desire for social distance may vary for different types of gambling. It would therefore in future studies be informative to compare the perspectives on gamblers of slot machines, casino gamblers, sports bettors and other gamblers. Greater familiarity with problem gambling and gamblers could effectively counteract stigmatisation.

There are several shortcomings in the present work, often caused by limited human and financial resources. For example, the use of different search terms, searches in other databases, and generally searches in other sources (e. g. manual searches) would have rendered further results. Due to time restrictions, the quality of the studies was not rated. The consideration of publications in other languages would also have been reasonable. Besides, the lack of formal assessment of intra- and inter-rater reliability can be criticised. Selection of the studies was made as team effort and all relevant decisions were extensively discussed. It would also be worth considering combining parts of the studies using meta-analytical statistical methods in the future.

Although a good number of studies focus on the public perception of persons with gambling problems, other aspects have been examined less well. Research on this topic started rather recently. In many countries, therefore, there is a lack of population-representative surveys on the perspective of gamblers. Moreover, it is noticeable that longitudinal studies are not available. These are however necessary to obtain insight into the dynamics of the phenomenon. It would also be interesting to monitor individual persons with gambling problems over a longer period of time with a qualitative research approach. Such a research project could provide important insights into the process of stigmatisation and in particular self-stigmatisation.

An interesting line of research is to look into the portrayal of persons with gambling problems on part of the media (press, official documents, films etc.). Since several studies have already been carried out, complementary research activities could start from a solid basis. The ways in which persons with gambling problems are portrayed in the media of different countries would also make an interesting topic of investigation, especially in countries where this issue has not yet been addressed.

Aspects such as the influence of the respondents ‘ sex and age on their attitudes have only been touched on briefly, if at all. Also, the influence of cultural aspects has hardly been taken into focus (Dhillon et al. 2011). In the same vein, there are only few studies empirically examining the public’s views and opinions on recreational gambling. Moreover, only few studies on the perspective of professionals on their clients exist, which is a clear indication that such research in this field should be intensified in the future, as the results are needed to advance stigma-free treatment (Schomerus 2017). This, in turn, would be necessary to ease access to treatment for those affected. To achieve this goal, targeted health policy measures should be enforced that systematically address stigmatisation. Individual harm might be diminished as a result, thereby improving public health.

## Footnotes

1 The term “person with gambling problem” will in the following be used for persons who experience problems with gambling, regardless of whether a diagnosis has been made or not.

2 The term “grey literature” refers to scientific works that are difficult to access via conventional access paths such as citation databases (e. g. Web of Science). Research reports are typical examples of grey literature. These works are often of the same quality as journal articles and should be taken into account in a comprehensive systematic literature review (Rothstein and Hopewell 2009).
